# A Real-Time Thermal Self-Elimination Method for Static Mode Operated Freestanding Piezoresistive Microcantilever-Based Biosensors

**DOI:** 10.3390/bios8010018

**Published:** 2018-02-28

**Authors:** Yu-Fu Ku, Long-Sun Huang, Yi-Kuang Yen

**Affiliations:** 1 Persol Technology Staff Co., Ltd., Tokyo 163-0451, Japan; yufuku15@gmail.com; 2 Institute of Applied Mechanics, National Taiwan University, Taipei 10617, Taiwan; lshuang@ntumems.net; 3 Department of Mechanical Engineering, National Taipei University of Technology, Taipei 10608, Taiwan

**Keywords:** thermal elimination, piezoresistive microcantilever, system miniaturization

## Abstract

Here, we provide a method and apparatus for real-time compensation of the thermal effect of single free-standing piezoresistive microcantilever-based biosensors. The sensor chip contained an on-chip fixed piezoresistor that served as a temperature sensor, and a multilayer microcantilever with an embedded piezoresistor served as a biomolecular sensor. This method employed the calibrated relationship between the resistance and the temperature of piezoresistors to eliminate the thermal effect on the sensor, including the temperature coefficient of resistance (TCR) and bimorph effect. From experimental results, the method was verified to reduce the signal of thermal effect from 25.6 μV/°C to 0.3 μV/°C, which was approximately two orders of magnitude less than that before the processing of the thermal elimination method. Furthermore, the proposed approach and system successfully demonstrated its effective real-time thermal self-elimination on biomolecular detection without any thermostat device to control the environmental temperature. This method realizes the miniaturization of an overall measurement system of the sensor, which can be used to develop portable medical devices and microarray analysis platforms.

## 1. Introduction

Piezoresistive microcantilevers (MCLs) were firstly proposed by Tortonese et al. in 1993 to minimize the entire system of an atomic force microscope, as well as to simplify the operation of the microscope [1]. Since then, piezoresistive MCLs have been developed and are widely used as pressure sensors [2], hygrometers [3], and accelerometers [4]. In biomedical sensing applications, Gunter et al., in 2003, detected aerosol-based and solution-based vaccinia virus by using embedded piezoresistive MCL sensors [5]. Wee et al., in 2005, reported a self-sensing, piezoresistive MCL sensor for the electrical detection of prostate cancer and cardiac disease markers [6]. Meanwhile, an MCL with an internal piezoresistive component has been utilized for an in situ, label-free, highly specific, and rapid DNA detection assay developed by Mukhopadhyay et al. in 2005 [7]. 

Conventionally, among these studies, the change in resistance is measured as an electrical signal by placing the piezoresistor in a Wheatstone bridge configuration. One sensing and one reference free-standing MCL beam with embedded piezoresistors as well as another two fixed resistors forming a Wheatstone bridge are used for eliminating environment disturbance. The sensing MCL is deposited with a sensitized film for detecting target analytes. Because of the unbalanced surface stress caused by the selective binding on the sensing MCL, the sensors are able to translate the bio-recognition events into nanomechanical deflection, leading to a resistance change proportional to the analyte concentration. Even so, the heterogeneous surface material properties for both beams contribute to the sensing and reference MCLs, which have independent deflection responses. The unexpected and disparate responses to most biological solvents or buffer fluids can affect the readout of real signals resulting in a problem of irreproducible results. In response to this issue, Yen et al. developed a single, free-standing, piezoresistive MCL sensor for detecting a biomolecule (C-reactive protein) with an improved precision [8]. However, the thermal-induced signal, including the temperature coefficient of resistance (TCR) and material bimorph effect, becomes a major issue in this configuration.

Although several groups proposed different methods [3,9,10,11] to reduce thermal effects on piezoresistive MCL sensors, most of them are based on double beam configurations and not applicable for bio/chemical detections. Thaysen used similar materials to make the thermal expansion coefficient of the two MCLs the same [12], but this method required an additional fabrication process, resulting in higher cost and lower yields. Differences in micromachining processes also made its design ineffective. Johansson et al. used SU-8 photoresist which is less sensitive to heat than the structure MCLs [11]. Although the signal of thermal effect can be reduced, the resistors are less robust because they are made of gold. The SU-8 cantilevers with integrated metallic piezoresistive readout are suitable for detection of small surface stress changes as long as the temperature is reasonably well controlled by the temperature control system. However, an additional temperature control system can only control the ambient temperature, but the temperature of the specimen that is injected into the sensor cannot be changed immediately because of the bimorph effect. These limitations will greatly reduce the practicality of piezoresistive MCL-based sensors. 

In this study, we propose a thermal self-elimination method for a biosensor based on a single, free-standing, piezoresistive MCL configuration. This strategy includes employing a fixed piezoresistor as an on-chip temperature sensor and an embedded piezoresistor in the MCL as a self-sensing temperature transducer. Through temperature gradient measurement, the relationship between these two resistances and the temperature is expressed as two quadratic functions. With the measurement of the temperature and through the interchange of these functions, we can eliminate the TCR and bimorph effect on the sensor. This method has an excellent temperature compensation ability in an environment with large temperature variations. Furthermore, in the absence of any thermostat device, the method was examined by using a piezoresistive MCL-based biosensor for C-reactive protein detections. The sensing capability of the biosensor and the feasibility of the system configuration were successfully verified.

## 2. Materials and Methods

### 2.1. Method of Thermal Self-Elimination

**Figure 1:**
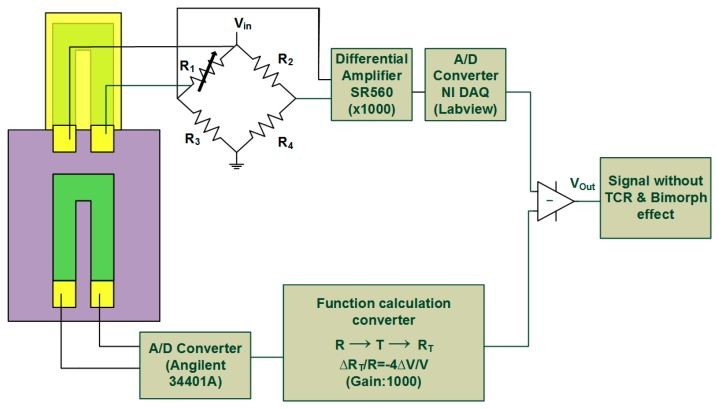
This scheme expresses the method of thermal effects elimination. By measuring the temperature-induced resistance changes of the fixed piezoresistor and suspended microcantilever (MCL), the relationships between temperature and resistance can then be represented as quadratic equations. After imputing these functions into our software, the system can automatically compute the real-time thermal effects reduction during the entire process of measurement.

This approach provides a device that includes an on-chip fixed piezoresistor that has *R_OC_* as the first resistance and an embedded piezoresistor of MCL as the second resistance *R_MCL_*, which are related to the temperature variation. A representation of this method is illustrated in Figure 1. First, the device was put in a temperature control system and then the temperature T adjusted, meanwhile it measured the change of resistance *R_fix_*. Second, by making the calibration curve of temperature and resistance according to this measurement, we can get the first quadratic function as following:

(1) $$R_{oc}=aT_{2}+bT+c$$

Similarly, by doing the same thermal cycle measurement of an embedded piezoresistor of MCL, we can obtain the second quadratic function:

(2) $$R_{MCL}=dT_{2}+eT+f$$

where parameters *a*–*f* can be calculated and are related to the materials. 

The Function (1) represents only the TCR effect but Function (2) indicates both the TCR and bimorph effect. The temperature *T* is the only shared parameter in these two functions. Therefore, in a real application, the resistance, *R_OC_,* is measured and substituted into Function (1), thus the real-time system temperature *T_sys_* is acquired. This is followed by substituting *T_sys_* into Function (2) and the temperature induced resistance *R_MCL_* is obtained. Next, the resistance *R_MCL_* can be converted to a voltage *V_Com_* by applying Wheatstone bridge principle. The converting process can be achieved by using a numerical calculation program as shown in Figure 1. Finally, after compensation, the output signal is determined by subtracting the converted voltage *V_Com_* from the voltage *V_MCL_* of sensing piezoresistive MCL. For easy understanding, the whole thermal self-elimination process can be expressed as following flow formula:

(3) $$R_{OC}\overset{f(1)}{\to }T_{sys}\overset{f(2)}{\to }R_{MCL}\overset{\frac{\Delta R}{R}=-\frac{\Delta V}{4V}}{\to }V_{Com}\overset{\left(V_{MCL}-V_{Com}\right)}{\to }V_{Out}.$$

The output voltage *V_Out_* is expected to present only the biological or chemical detection signal. 

As shown in Figure 1, the signal of resistance change of the sensing MCL was obtained through the Wheatstone bridge and then sent to the two ends of the preamplifier, SR 560, which can amplify the signal 1000 times. Next, a 0.1 Hz low-pass filter was used to process the signal for computer reading. In addition, the signal of resistance change of embedded piezoresistor was acquired by using Agilent 34401A meter (Agilent technologies, Inc., Santa Clara, CA, USA) which can convert the analog signal to a digital signal (accuracy 0.1 ohms). Then, the corresponding compensation voltage was obtained by software conversion through the Wheatstone bridge principle. For the calibration, the temperature was controlled and monitored. Therefore, the signals of voltage change of the free-standing sensing MCL and fixed piezoresistor can be obtained at the same time and through the reduction of the two signals to compensate the thermal effect on the sensor. 

### 2.2. Design and Fabrication of Piezoresistive MCL-Based Sensor

**Figure 2:**
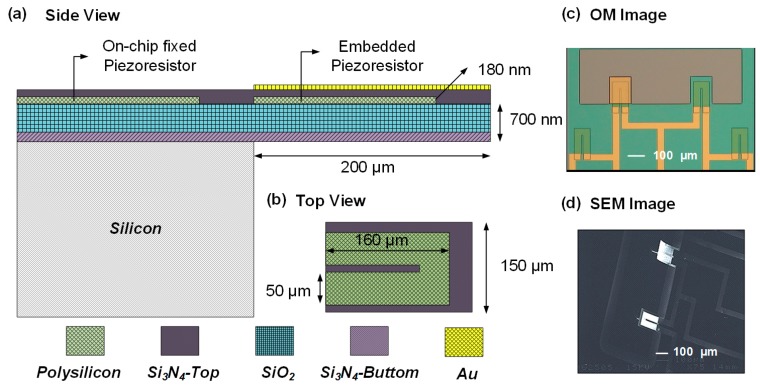
(**a**) The side view and (**b**) the top view of the design of piezoresistive MCL sensor chip; (**c**) an OM (optical microscopy) image of the chip layout and (**d**) an SEM image of a piezoresistive MCL sensor chip after complete fabrication process.

Due to the need for leakage and internal stress balance, the piezoresistive MCL used in this study was a composite structure composed of five layers. The material and fabrication parameters of each layer are listed in Table 1. The size of the MCL was designed as 200 μm in length, 150 μm in width, and 1.265 μm in thickness, which is shown as Figure 2a,b. To fabricate an MCL-based sensing chip, a 100 nm of silicon oxide as a protective layer was first deposited on a 500 μm p-type <100> silicon wafer by using plasma enhanced-chemical vapor deposition (PECVD). In order to maintain the piezoresistive layer away from the neutral axis of MCL, a 600 nm of low-stress silicon nitride was further deposited by using low-pressure chemical vapor deposition (LPCVD). Next, the piezoresistive layer of MCL was formed by depositing a 180-nm layer of polysilicon on silicon nitride by LPCVD at 620 °C and was immediately followed by the boron implantation at 30 keV with a dose of 3.21 × 10^19^ cm^𢀒3^. After implantation, the annealing process was executed at 1050 °C for 30 min to increase the formation of a monocrystalline silicon and reduce the density of lattice defects, which can allow barrier down, and then enhance the conductivity. 

Subsequently, the shape of piezoresistor was patterned and defined on a polysilicon layer by using reactive ion etching (RIE). A 16/180 nm of Cr/Au layer was then deposited using an e-beam evaporator and defined to connection wires and bonding pads by a wet etching process. After this, 350 nm of silicon nitride was deposited as an insulation layer by employing PECVD. To this point, all layers of the MCL composite structure were processed completely and followed by using RIE to define the shape of MCL. Before the final releasing step, a 35 nm gold layer was deposited for the immobilization of chemical probes. A deep-reactive ion etching (DRIE) was initially used to etch the silicon substrate, and the piezoresistive MCL was then released by etching the silicon substrate in a KOH solution. The OM and SEM image of the complete piezoresistive MCL chip is shown in Figure 2c,d.

### 2.3. Microfluidics and Electronic Readout System

The microchannel was a crucial component in this study, which was necessary to provide a stable flow field and appropriate environment for measurement. The microchannel was designed to be 2400 μm wide, 1000 μm long, and 100 μm deep. The flow rate in the channel was 0.6 mL/h and therefore the flow in the channel can be considered a laminar flow. Polydimethylsiloxane (PDMS) was chosen to produce an inert and non-toxic micro-chamber. 

**Figure 3:**
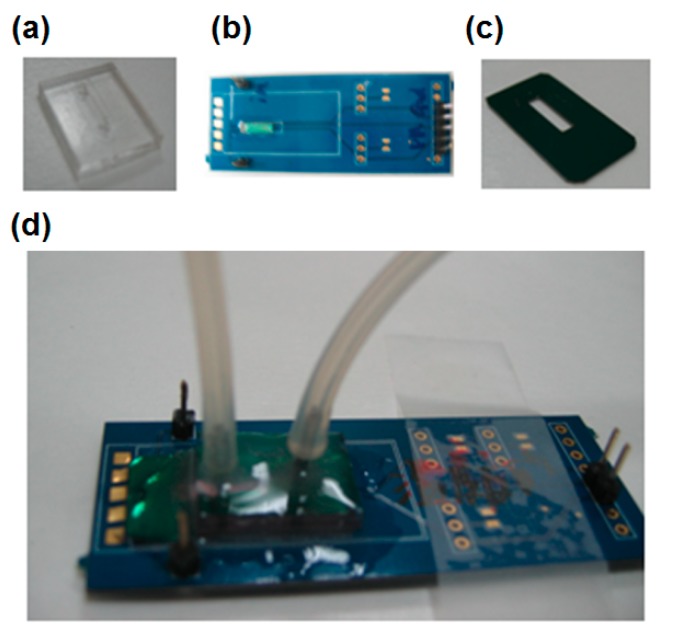
Components and combination of the microfluidics and PCB-based readout system (**a**) PDMS microchannel (**b**) Designed PC-board (**c**) Silicon channel base (**d**) Assembly completed figure.

The PCB-based chip carrier was designed for the signal readout system, where it can integrate the MCL sensing chip with microfluidics and electronic devices as shown in Figure 3. The piezoresistive MCL sensing chip was glued and wire bonded on a PCB. Then exposed contacts were protected and insulated by using silicone glue (Dow Corning^®^ 3140, Dow Corning Corporation, Hillsdale, MI, USA). In order to connect the PCB with the PDMS microchannel, a silicon bottom plate had previously been glued on the PCB. Finally, the microchannel was glued onto the silicon plate to complete the package of the sensing chip. The stainless-steel tubes were inserted into the PDMS microchannel as an inlet and outlet, as well as pin sets were soldered for wire connections with a measurement meter. 

### 2.4. Surface Modification

**Figure 4:**
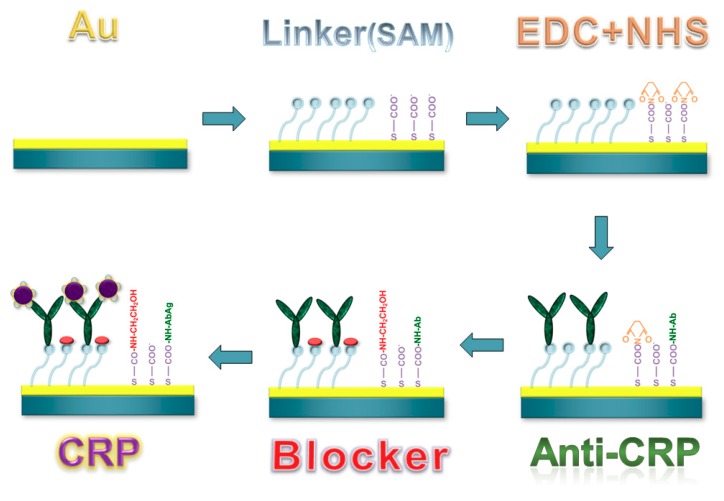
The surface functionalization and the bioassay of the MCL-based biosensor.

To functionalize the sensing layer of the piezoresistive MCL chip (Figure 4), the gold layer surface must be well cleaned. The chip was sequentially immersed in solutions of 0.1 N hydrochloric acid and 0.1 N sodium hydroxide, acetone, and ethanol. Each cleaning step was processed for three minutes and then washed three times with deionized water before the next step. To form the bio-linking self-assembled monolayer (SAM) on the gold layer, the MCL chip was incubated in 100 mM 8-mercaptooctanoic acid (Sigma-Aldrich Co., St. Louis, MO, USA) solution for 24 h to achieve a dense alignment. Then the chip was rinsed with pure ethanol followed by immersion in a mixed solution of EDC (N-(3-Dimethylaminopropyl)-N’-ethylcarbodiimide hydrochloride) and NHS (N-Hydroxysuccinimide) (Sigma-Aldrich Co., St. Louis, MO, USA) for 30 min to activate the functional group of the SAM. The solution was 400 mM EDC mixed with 100 mM NHS in 1:1 volume ratio. After activation, a 100 μg/mL of probing C-reactive protein (CRP) antibody (Sigma-Aldrich Co., St. Louis, MO, USA) solution was injected into the microchannel and then incubated for 3 h. After the antibody was immobilized, 1 M ethanolamine was added to passivate the unbinding linker of SAM so as to achieve better specificity. As a result, the surface of the piezoresistive MCL sensor was completely functionalized and ready for further measurement. 

## 3. Results and Discussion

### 3.1. On-Chip Piezoresistive Thermometer

**Figure 5:**
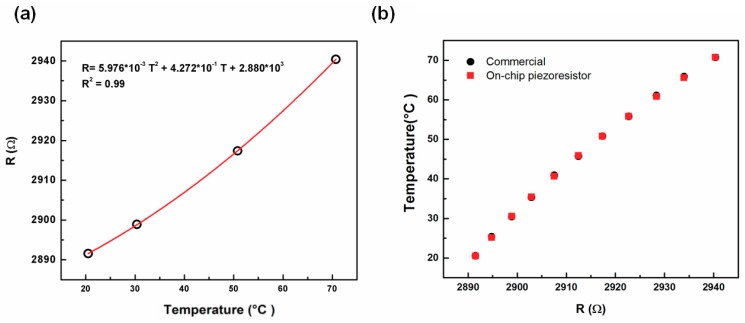
(**a**) The relationship between the relative changes in resistivity of TCR (temperature coefficient of resistance), and changes in temperature of the unreleased on-chip piezoresistor from 20 °C to 70 °C; (**b**) The comparison between on-chip piezoresistive thermometer and commercial thermometer.

The relationship between the relative changes in the resistivity of TCR and temperature of the embedded fixed piezoresistor from 20 °C to 70 °C is shown in Figure 5a. This relationship can be fitted to a quadratic equation. From the results, the on-chip fixed piezoresistor can be utilized as a thermometer to determine real-time temperature in the microchannel through a mathematical transformation. The result was compared with the temperature measured by a commercial thermometer shown in Figure 5b. The difference of the measured temperature between the two devices was 0.2% which confirmed the reliability of the on-chip fixed piezoresistor as a thermometer. 

**Figure 6:**
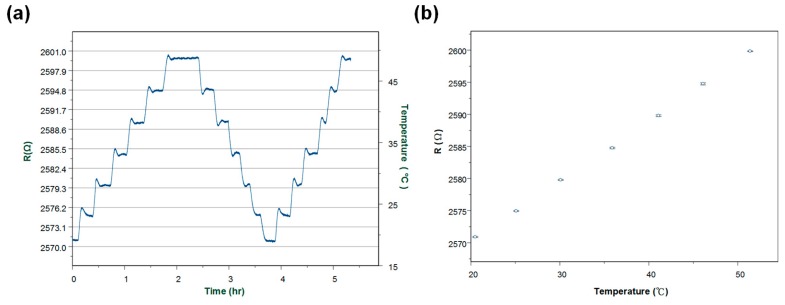
(**a**) Measurement of resistance change in doing up and down sweeps of the temperature for 5 h; (**b**) The signal of resistance change with respect to temperature variation in sweeping temperature control measurement.

Moreover, the hysteresis for temperature measurements was studied. Through at least three experiments which lasted for 5 h (Figure 6a), it was found that resistance change was about ±1 ohm with respect to a temperature variation of ±0.2 °C in doing up and down sweeps of the temperature. During various measurements, the coefficient of variation was 0.04% as shown in Figure 6b.

### 3.2. Thermal Effects Elimination

By using the same calibration method as in the previous section, we can obtain two quadratic functions in the same form using Functions (1) and (2), which are

(4) $$R_{oc}=1.422\times 10^{-3}T_{2}+8.428\times 10^{-2}T+2719$$

(5) $$R_{MCL}=1.085\times 10^{-3}T_{2}+9.861\times 10^{-1}T+2667$$

When measuring the resistance change in *R_OC_* of the on-chip piezoresistor, the real temperature can be obtained from (4), followed by substituting the temperature into (5) and getting temperature-induced resistance change *R_MCL_* of the freestanding piezoresistive MCL. This two-step mathematical transformation was automatically performed by our LabVIEW software. The resulting value of the resistance change was output as the compensation voltage through the Wheatstone bridge and the amplifier circuit. The output signal was finally obtained by subtracting the compensation voltage from the induced voltage of sensing piezoresistive MCL.

**Figure 7:**
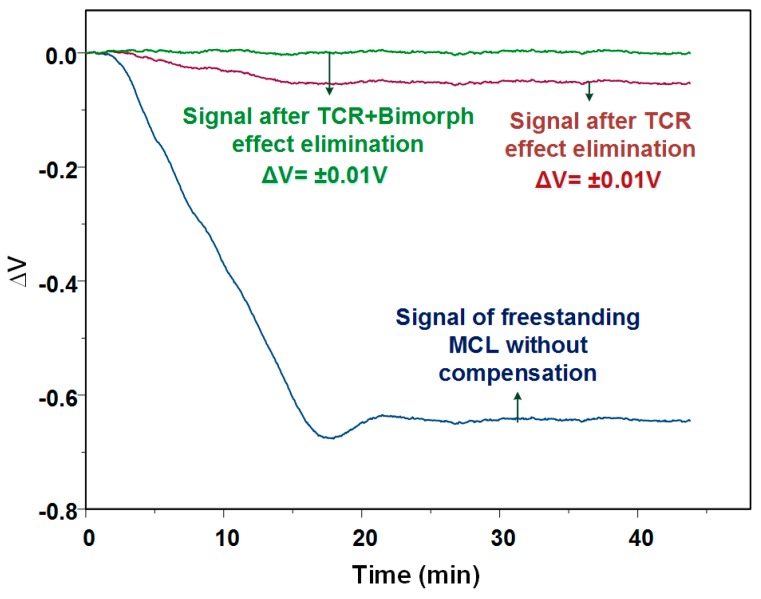
The output signal before and after applying the thermal self-elimination method, which the input voltage was 0.3 V. The voltage change induced by TCR was about 23.6 μV/°C while the bimorph effect caused a voltage change of 2 μV/°C.

Figure 7 shows the output signal before and after thermal elimination, when the temperature increased form 13.6 °C to 40.7 °C. The input voltage for the Wheatstone bridge was 0.3 V. It was found that the voltage change caused by the total thermal effect was about 23.6 μV/°C, while the bimorph effect produced a change of 2 μV/°C. It revealed that the output signal induced by TCR was about ten times larger than that induced by the bimorph effect. The final output signal also showed that the effective elimination of thermal effects by applying this method. If recording the signal after thermal effect elimination for analysis, it can be found that the output voltage variation was 0.3 μV/°C, which was almost two orders of magnitude less than it was before processing the thermal elimination method. 

We propose several reasons to explain the existing signal noise. First, since the temperature control system used in this experiment had ±0.2 °C of control error, the temperature calibration error resulted in a small error in the temperature calibration curve. The use of a more accurate temperature control device for measurement can improve accuracy. Another approach is to take the measured temperature error into the correction of the quadratic function in order to obtain more precise parameters *a*–*f*. Second, the noise came from an electric circuit and the reading error induced by the electrical instrument. Using an amplifying with low-pass filtering circuit for the electrical readout may decrease spurious signals. 

**Figure 8:**
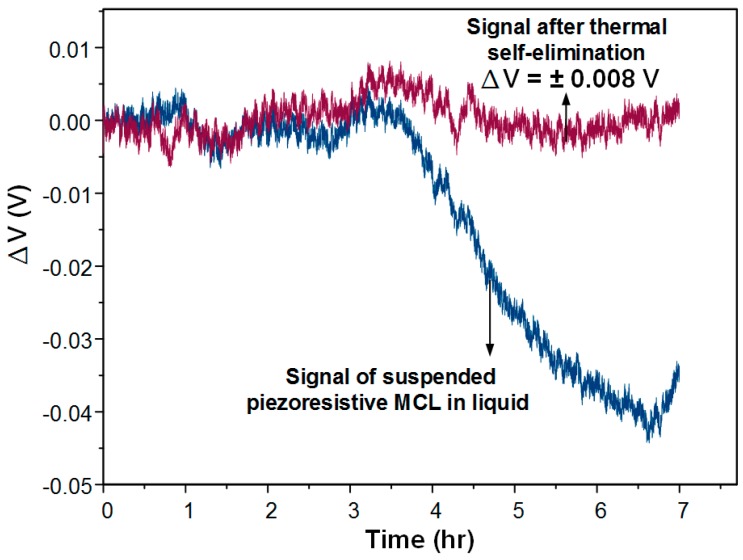
The compensated signal and the original signal of the MCL based sensor placed in liquid. (flow rate: 0.6 mL/h).

The piezoresistive MCL sensor was further tested in an aqueous environment for a long time (7 h). The solution injected was PBS buffer and the flow rate was controlled at 0.6 mL/h. The result shows that the real-time compensation method still worked in the fluid, as shown is Figure 8. We found that the signal change from the freestanding MCL was 0.04 V at 3 h induced from 1.8 °C of room temperature change. The signal after compensation was maintained at around 0 V; however, it was not affected by the temperature-induced signal drift. The noise of the signal can result from flow field oscillations such as small bubbles on the interface of the fluid and surface of the MCL. This was a real response signal of the sensing MCL having nothing to do with the temperature effect.

### 3.3. C-Reactive Protein Detection with Real-Time Thermal Effects Elimination

**Figure 9:**
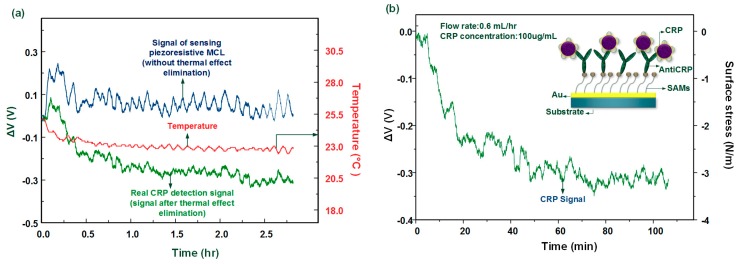
(**a**) The signals before and after using thermal elimination method to detect CRP at a 100 μg/mL concentration at room temperature; (**b**) The corresponding signal of the surface stress changes for detecting 100 μg/mL CRP.

In protein detection, 100 μg/mL of the CRP solution (pH 7.2) was injected into the MCL sensing chip through the microchannel. The whole measurement was processed at room temperature without any temperature control system. The result shows that the proposed method successfully improves the piezoresistive MCL sensor for biomolecule detection as shown in Figure 9a. The original signal without thermal effect elimination varied around 0 V due to the thermal induced signal coupled with the biochemical-induced signal. Such signals cannot provide any information for the existence of CRP. After applying the real-time thermal self-compensation method, the real tendency of the signal of CRP captured by anti-CRP was expressed. 

Moreover, from Figure 9a, we found that the system can successfully measure the biological sensing signal as well as the temperature signal. We found that the ambient temperature decreased by about 2 °C, which caused the corresponding TCR and bimorph effect, leading to a false readout signal. The voltage change signal of the antigen–antibody interaction can be converted into a value for the change of the surface stress [13] as shown in Figure 9b. In CRP detection, the signal indicated that the MCL deflects upward and the surface stress change was about −3.1 N/m (compressive surface stress). This biologically induced surface stress change was consistent when compared with our previous work [8], which used a large external temperature control device to maintain the environment temperature. Therefore, this method was shown to successfully remove the thermal effects when using a freestanding MCL-based sensor for biomolecular detection. The whole system was miniaturized onto a chip consisting of sensing and fixed MCLs combined with the computing software, which was used to develop the multi-functional MCL array. 

To improve performance of this method, we propose several ideas. First, in the current design, the position of the on-chip fixed piezoresistor is 100 μm from the suspended sensing MCL leading to a temperature difference between the two devices in the liquid environment. We suggest that the two devices be placed side by side to decrease the differences in temperature-sensing. Second, the whole process in the experiment is necessary to avoid bubble generation in the microchannel, since bubbles can greatly disrupt the flow field in the reaction area, resulting in measurement interruption and signal fluctuation. Moreover, before the injection of each sample, the tubing should be cleaned in order to avoid contamination with residue from the previous sample. 

## 4. Conclusions

In this study, a feasible thermal self-compensation method for a piezoresistive MCL-based biosensor was proposed. This is the first report of the method of bimorph effect elimination. The apparatus required only an MEMS fabricated chip including an unreleased piezoresistor and a freestanding MCL, together with the computing software. The calibration of the relationship between the resistance change and temperature was needed before applying the method to any measurement. The whole system was miniaturized and simplified. Good performance of thermal effect reduction was achieved in air and liquid environments with a large temperature variation over a long time. The method was determined to be capable of small signal measurement in biomolecular detections. This achievement opens up a wide array of possibilities for the real application of electrical readout-based MCL biosensor. 

## Figures and Tables

**Table 1:** Material and fabrication parameters of MCL.

Layers	Materials	Thickness (nm)	Micromachining Process
1	Gold	35	E-beam Evaporator
2	Silicon Nitride	350	PECVD
3	Polysilicon	180	LPCVD
4	Silicon Nitride	600	LPCVD
5	Silicon oxide	100	PECVD
